# Understanding stakeholder perceptions of the implementation and development of an emerging advanced nursing role in cancer care: a Swedish concept mapping study

**DOI:** 10.1136/bmjoq-2026-004573

**Published:** 2026-11-06

**Authors:** Ellen Brynskog, Maria Larsson, Mikael Holtenman, Kaisa Bjuresäter, Frida Smith

**Affiliations:** 1 Department of Health Sciences, Karlstads University, Karlstad, Sweden; 2 Regional Cancer Centre West, Gothenburg, Sweden; 3 Institute of Clinical Sciences, Sahlgrenska Academy, Gothenburg, Sweden

**Keywords:** Nurses, Management, Governance, Neoplasms, Quality improvement

## Abstract

Nursing roles in cancer care are becoming increasingly autonomous and complex. In the Swedish setting, the role of contact nurses in cancer care (CNCC) exemplifies this movement and has been implemented and developed to enact person-centred care and improve quality. However, the scope and organisation of CNCC services vary across Sweden. The manner in which the implementation and development processes have unfolded within the governance and leadership structures of the cancer sphere is important to review in this regard, to effectively develop the role further. The aim of the study was to investigate stakeholders’ perceptions regarding what factors have impacted the implementation and development of the CNCC role in Swedish cancer care.

A five-step concept mapping study was conducted, including preparation, generating ideas, structuring the ideas, concept mapping analysis and interpretation. Participants represented four stakeholder groups: local leadership and governance, regional and national leadership and governance, organised advocacy groups and academia. 83 persons completed the idea generation phase, 29 completed the sorting phase, and 25 persons participated in the interpretation phase.

The concept map consists of 89 unique ideas in seven clusters labelled as follows: Understanding and delineation of the role, Variance in resource allocation and prioritisation, The importance of national governance, The importance of local governance, The legitimacy of nursing in the complex processes of cancer care, Motivation and support for competence development and Patients’ needs and position. Together, they depict how the negotiated nature of the implementation and development process on several structural levels has impacted the role. Role delineation difficulties emerged as a central topic. Leadership commitment and larger trends in nursing and healthcare also seem to have impacted the development and implementation of the CNCC role in Swedish cancer care. Insights from this study can inform the continued development of the role in Swedish settings and beyond.

**WHAT IS ALREADY KNOWN ON THIS TOPIC**

- Contact nursing services in Swedish cancer care have the potential to bring much value to those affected by cancer, but the scope and organisation of services vary significantly across settings.

**WHAT THIS STUDY ADDS**

- By investigating the understandings of stakeholders (such as managers, civil servants, advocacy groups and academics) that shape the conditions under which contact nurses in cancer care (CNCCs) and patients interact, a clearer picture of the effect that management and governance have had on the role of CNCCs emerged. The results depict the intricate dynamic between formal and informal governance structures and leadership at the micro, meso and macro levels as well as how larger trends in nursing and healthcare have played into the development of the CNCC role.

**HOW THIS STUDY MIGHT AFFECT RESEARCH, PRACTICE OR POLICY**

- Insights from this study can inform the continued development of the CNCC role and services. In a more general sense, it serves as a case study regarding what factors shape major improvement and development endeavours concerning advanced nursing roles in cancer care.

## Background

 Cancer care is a complex field shaped by multiple disciplines, actors and processes. Current challenges include inadequate coordination and communication between healthcare actors and unmet psychosocial and informational needs among patients.^1^ In addressing these gaps, nursing services play a central role. Over recent decades, a substantial development regarding scope, degree of autonomy and complexity of nursing services in cancer care has taken place to improve quality of care for those affected by cancer.^2^ Examples of this development are the roles of oncology nurse navigator and cancer clinical nurse specialist, often requiring a minimum of a master’s degree.^3^

In Sweden, this evolution is reflected in the emergence of the CNCC role. CNCCs are meant to function as the patients designated health professional and primary point of contact. A national role description, approved by the Confederation of Regional Cancer Centres, identifies four core areas of responsibility: (i) coordination and coherent care processes, (ii) availability and continuity, (iii) patient participation and influence throughout the care process and (iv) assessment and management of individual care needs.^4^ The national cancer strategy states that implementation of the CNCC role is important to facilitate person-centred care,^5^ a care philosophy and ethic that has gained global recognition, emphasising people’s experiences, dignity and humanism in care delivery.^6^ Unlike some of the role’s international counterparts, the CNCC role is not formally regulated with regard to educational requirements (beyond a bachelor’s degree in nursing) or qualifications based on clinical experience. Thus, a wide variety of services and arrangements fall under the term CNCC.^7 8^ Although the CNCC role does not correspond to advanced practice nursing in a strict regulatory sense, it reflects broader international developments towards increasingly autonomous, coordinated and specialised cancer nursing roles. The CNCC role is therefore considered as an emerging advanced nursing role within Swedish cancer care.

The assessment of the emerging nursing roles in cancer care has often focused either on patients’ experiences or outcomes^9–13^ or on various aspects of the nurses’ experiences and insights.^14–19^ Both perspectives can be understood as expressions of outcome or processual dimensions of quality.^20^ Findings from such assessments in the Swedish context include that CNCCs struggle to provide cancer rehabilitation according to national guidelines^15^ and that they experience their role as extensive, yet unclear and unsupported.^14 21^ Previous research on advanced nursing roles in cancer care from Swedish, European and North American settings has shown that these roles and services have the potential to improve quality of care for people affected by cancer.^3 7 22–24^ Less attention has been paid to how governance structures, leadership processes and organisational actors shape the implementation and development of such roles. This perspective has also been noted as a knowledge gap in healthcare quality research in a more general sense.^25^ This study uses the CNCC role and the Swedish context as a case to explore perceptions and insights of key stakeholders involved in its implementation and development. As the governance of the healthcare sector is multi-layered, the structural domain of healthcare can be reviewed from different angles ranging from the micro (local governance and leadership capacities) to the meso (regional) and macro (national and international) governing structures and processes shaping healthcare practices. To obtain a comprehensive understanding, it is essential that stakeholders representing these different levels are included. The aim of the study was to investigate stakeholders’ perceptions of what factors have influenced the implementation and development of the CNCC role in Swedish cancer care.

## Method

Concept mapping (CM) is a participatory method where qualitative and quantitative elements are integrated through a structured, stepwise process. It allows facilitation of diverse perspectives, and participants are engaged in both generating and analysing data.^26 27^ The outputs are visual representations – concept maps – of the participants’ insights, depicting their relatedness. CM has repeatedly been applied in healthcare evaluation and development studies^28–32^ and was deemed a suitable methodological choice given the aim. The six-step process encompasses: preparation, idea generation, structuring of ideas, analysis, interpretation and utilisation.^27^ The first five steps are reported in the current study, whereas the utilisation phase will be conducted outside the study format as the research aim is of an evaluative rather than action-oriented nature. The study was conducted between September 2025 and February 2026. Figure 1 describes the process. The manuscript is annotated in accordance with the newly developed ConMapT guideline^33^ (see online supplemental appendix 1).

**Figure 1:**
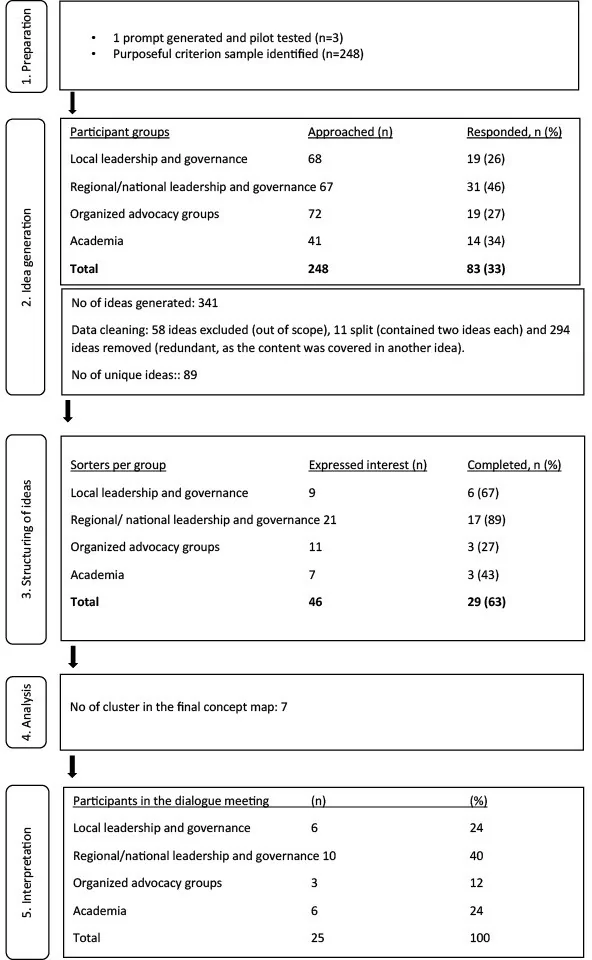
Flowchart of the study process.

### Patient and public involvement

The CM methodology heavily emphasises the involvement of stakeholders throughout the research process. Previous insights from stakeholders guided the design and development of the study, and stakeholders both generated and analysed data. For further detail, see figure 1 and Phase 1–5, below.

### Setting

#### The QOPCoN project

This study is part of the research project ‘Quality of care and Organisational Prerequisites for Contact Nurses’ (QOPCoN). The project defines organisational prerequisites as structural attributes of a healthcare organisation that influence the processes and outcomes of care by impacting staff’s ability to provide high-quality care. Key dimensions include leadership, workplace culture, workload and tools for work, formal competence, organisational commitment to healthcare improvement and organisation of work. Central actors shaping these prerequisites are formal and informal leaders, boards administrators and civil servants at the micro, meso and macro levels.

#### The Swedish healthcare setting

The governance of the Swedish cancer care sector is multi-layered, decentralised and mainly public. Sweden is divided into 21 politically governed regions with far-reaching authority over the organisation and delivery of care. They cooperate through six clusters (healthcare regions), to optimise the utilisation of resources and have established a national system for knowledge-driven management. It is a collaborative, non-mandatory system that produces knowledge support and facilitates open comparison and monitoring.^34^ In cancer care, this management system is particularly developed through civil servant organisations called Regional cancer centres.^35^ The state provides frames for healthcare through legislation, fiscal support, and supervising and expert authorities.^36^

Specialised cancer care is provided at university, county and smaller county hospitals. CNCCs operate at all of these, principally in outpatient clinics.^8^

### Phase 1: preparation

A prompt (an unfinished sentence) was generated, aiming to obtain multiple and diverse ideas from participants.^27^ Three potential participants were recruited to pilot-test the prompt regarding comprehensibility. After adjustments, the prompt read:

Something that has influenced the development of the contact nurse role (positively or negatively) in Swedish cancer care is…

Although the aim of the study concerns both the implementation and the subsequent development of the role, the term ‘development’ was deemed sufficient for the prompt, given how the term is used in everyday Swedish language.

A purposeful criterion sample^37^ from four stakeholder groups was invited to participate: Local leadership and governance, Regional and national leadership and governance, Organised advocacy groups and Academia. These groups were selected to ensure representation from relevant stakeholders across the multi-level governance structure of Swedish cancer care (Local leadership and governance and Regional and national leadership and governance), as well as stakeholders influencing these structures (Organised advocacy groups and Academia). Table 1 describes the motive and constitution of the stakeholder groups.

**Table 1:** Overview of stakeholder groups and their reason for inclusion

Stakeholder group	Examples of roles of invited stakeholders	Reason for inclusion of the group
Local leadership and governance	Unit and operations managers and strategists in cancer care, comprehensive cancer centre representatives.	Responsible for operational implementation and development.
Regional and national leadership and governance	Civil servants at regional cancer centres, regional cancer coordinators, civil servants at national authorities.	Responsible for governance and policy development and implementation support.
Organised advocacy groups	Representatives of healthcare professionals’ associations, patient associations and councils.	Advocacy organisations that can influence governance and policy development.
Academia	Researchers or faculty educating CNCCs and/or conducting research concerning the CNCC role in some regard.	Responsible for knowledge production and education to support practice and policy development.

CNCC, contact nurses in cancer care.

Eligibility was assessed by the research group in relation to the study aim and the selected stakeholder groups. Efforts were made to ensure all groups were sufficiently represented in the approached sample. Eligible participants (n=248) were identified through an environmental scan, using the research group’s professional network and relevant web pages. Some identified individuals had held positions in several stakeholder groups and were placed in the group of their most recent relevant role. Snowball sampling was also employed, as some forwarded the study invitation to colleagues (for example, due to long-term absenteeism, or because they considered a colleague more suitable). An email was sent to the eligible participants, with information about the study and a link to a digital survey. Three reminders were sent. No demographic data was collected from participants.

### Phase 2: generating the ideas

Participants were asked to complete the prompt two to five times. These responses are referred to as ‘ideas.’^26^ At this stage, 341 ideas were provided by 83 participants (response rate 33%). Idea synthesis was thereafter supported by IdeaDistiller, a recently validated AI-assisted CM tool based on semantic clustering and a human-in-the-loop approach. The tool uses BERTopic, sentence embeddings and hierarchical clustering to organise semantically related ideas, but does not replace researcher judgement. All outputs were reviewed and validated by the research team before further analysis.^38^

Thereafter, statements deemed to express the same underlying idea were merged, while compound statements containing more than one concept were split into separate statements. Statements outside the scope of the focus prompt were excluded (n=58). Minor grammatical and spelling corrections were made to improve readability while retaining participants’ original wording and meaning whenever possible. Through this iterative synthesis process, the initial 341 ideas were reduced to 89 unique ideas, a suitable data set for the subsequent study stages.^26^ A complete list of the unique ideas is available in online supplemental appendix 2.

### Phase 3: structuring the ideas

All participants in phase 2 were invited to participate in phase 3. Forty-four people—plus two individuals who were recruited for this stage (n=46)—agreed and received an email with a link to an internally developed sorting software where the 89 unique ideas had been imputed. Participants were instructed to group ideas based on how they perceived themes and relatedness, with no specification regarding how many groups to create. They were also asked to name groups. Three reminders were sent. 29 persons (63%) completed the sorting task. The number of groups produced by sorters ranged from 4 to 14 (mean 7.9, median 7).

### Phase 4: concept mapping analysis

Multidimensional scaling (MDS) was used to merge all the sortings into a two-dimensional map, where each point represents a unique idea, and the distance between points indicates how often participants have grouped ideas together.^26^ A stress value was computed to evaluate the degree of distortion introduced by the MDS. The obtained stress value, 0.27, falls below 0.392, which is the cut-off given the number of items and dimensions, suggesting that the two-dimensional solution provides a satisfactory representation of the data.^39^

A hierarchical cluster analysis (HCA) was conducted using Ward’s minimum variance method and Euclidean distance measure. The analysis was based on all 89 two-dimensional points from the MDS output. Solutions ranging from 5 to 15 clusters were produced and scrutinised by the research group to establish the solution representing the most coherent content per cluster, balancing the need for both clarity and detail in regard to the study aim.^40^ The seven-cluster solution was deemed most appropriate. The tentative seven clusters were labelled descriptively through discussion, based on the labels generated by sorters.^27^

Quantitative analysis was conducted in R (R core team V. 4.5.0).

### Phase 5: interpreting the map

^27^ All participants from the idea generation phase (n=89) were invited to a 2 hour digital dialogue meeting after the finalisation of the MDS and the HCA. 25 persons participated. The tentative concept map was presented, after which group discussions under the leadership of authors (EB, ML, KB and FS) were facilitated to ensure all participants could voice their opinions and ideas, thus managing the risk of power imbalances. Participants from different stakeholder groups were mixed and the discussions gravitated around the validity of the tentative map and deepened insights regarding one or two clusters per group. As a dialogue-based prioritisation exercise, participants were asked to collectively reflect on which idea(s) in the respective clusters were the most significant contributors to the development of the CNCC role. These ideas are presented in figure 2.^27^ Cluster seven was renamed as a result of the discussions in the dialogue meeting.

**Figure 2:**
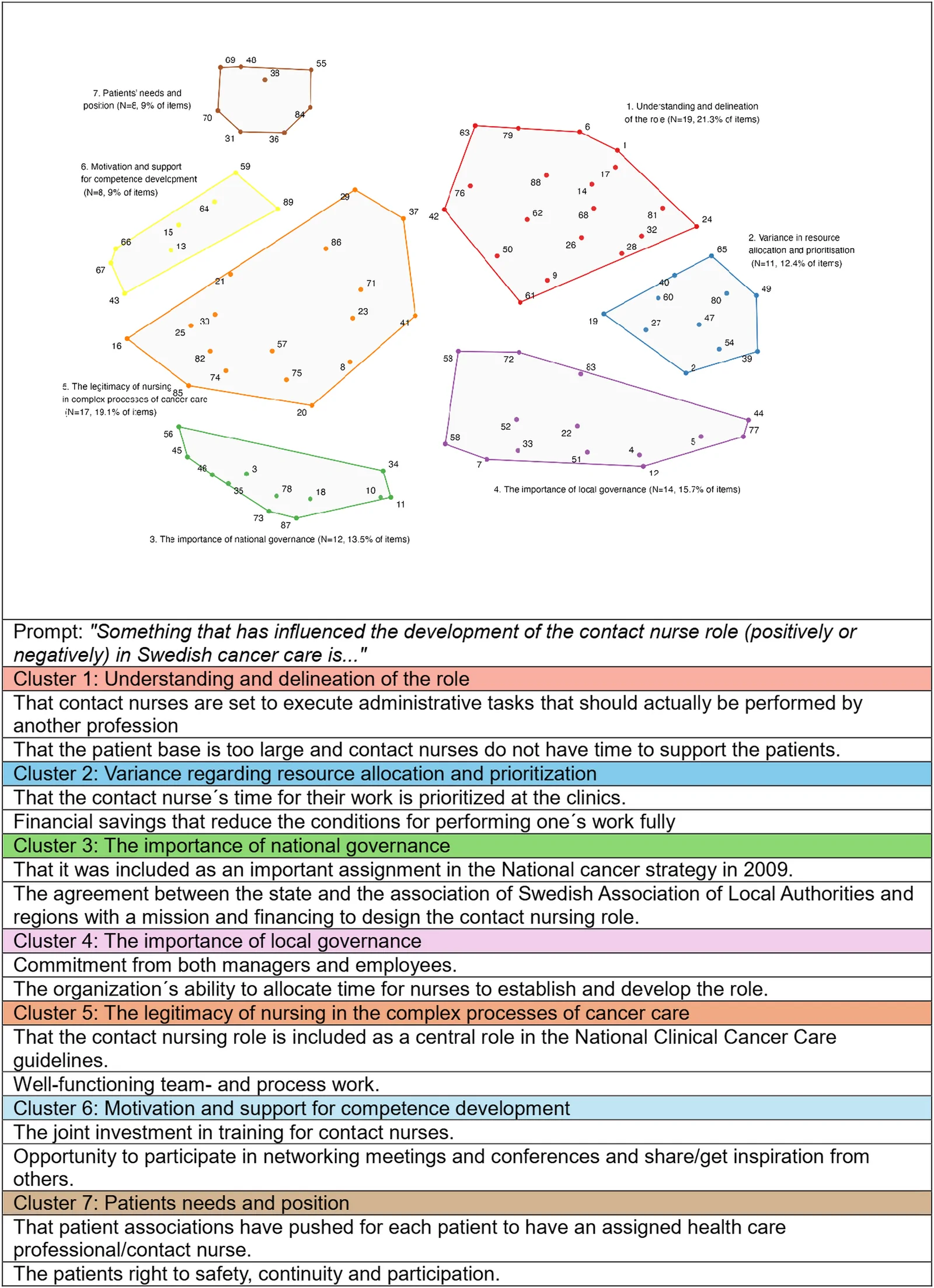
Concept map depicting the seven clusters and the number and percentage of original ideas included in each cluster. Each dot in the map represents one unique idea. Ideas highlighted by participants in the dialogue meeting as particularly impactful for the implementation and development of the CNCC role are described below the map. CNCC, contact nurses in cancer care.

After the dialogue meeting, the research group collectively reviewed and discussed the data from all phases, including dialogue meeting transcripts and recordings.

### Description of the final concept map

The stepwise data collection was consolidated into a concept map consisting of the 89 unique ideas distributed in seven clusters. The clusters were labelled as follows: understanding and delineation of the role; variance in resource allocation and prioritisation; the importance of national governance; the importance of local governance; the legitimacy of nursing in the complex processes of cancer; motivation and support for competence development and patients’ needs and position. All seven clusters, together with the ideas highlighted as particularly significant by participants in the dialogue meeting, are presented in figure 2.

#### Cluster 1: understanding and delineation of the role

This cluster encompasses participants' ideas and insights regarding how the lack of clarity regarding the CNCC role has impacted its implementation and development. An ongoing negotiation for clarification could be identified at several structural levels and across groups of actors. On the micro level, concerns about scope of practice and delineation in relation to other team members and professions were described by participants. As the role was only *suggested* through the national cancer strategy (and not mandated through the usual decision-making lines), it was more difficult to achieve consensus across management levels on the what, how and why of the role at micro, meso and macro governance levels. Participants expressed that the scope and purpose of the role are often perceived as clear by CNCCs, but that it tends to become organisationally fuzzy, partially because CNCC is not (yet) a protected or formal title.

#### Cluster 2: variance in resource allocation and prioritisation

This cluster highlights participants' perceptions of how operations have emphasised the implementation and development of the CNCC role to varying degrees. Participants traced the reasons for this partially back to cluster one; a lack of understanding of the role and its formalisation was described as a focal factor, shaping operations’ ability and willingness to allocate resources to the role, not least in terms of providing CNCCs with sufficient time to properly perform it. In the absence of sustained organisational support to implement and cultivate the role, the commitment of CNCCs and first-line managers was described as decisive.

Participants frequently described clusters one and two as particularly important for understanding the implementation and development of the CNCC role. The research team thus interpreted these clusters as central within the overall concept map.

#### Cluster 3: the importance of national governance

The style and scope of governance from macro-level national actors were emphasised by the participants. The launch of the Swedish national cancer strategy in 2009 was described as a catalyst for the role. It had the appearance of a top-down decree, but our findings indicate that the subsequent national governance strategy has been characterised by volunteerism and pragmatism, rather than requirements, control and compliance. The formation of the six regional cancer centres and their efforts to influence and support operations were described by participants as impactful for the development of the role, but not as optimal from an efficacy perspective, as they operate outside of the formal decision-making lines.

#### Cluster 4: the importance of local governance

The local (micro/meso) management structures were described by participants as a decisive gatekeeping level, impacting the degree to which CNCC services have been allowed to flourish and develop, with a clear connection to clusters one and two. However, it was highlighted that local governance structures and leaders are bound to deliver on goals set at higher structural levels. Thus, first-line managers’ understanding of the benefits of the CNCC role and their willingness and ability to communicate these benefits upwards within the organisation were described as pivotal.

#### Cluster 5: the legitimacy of nursing in the complex processes of cancer care

This cluster features ideas that focus on how nursing more broadly is viewed and valued at different structural levels in cancer care. Formal recognition of the role in national clinical care guidelines and research was described as meaningful both for strengthening CNCCs in their role in the team and for facilitating access to resources and competence development.

The lack of formal competence requirements and the under-utilisation of evidence from nurse-led clinics internationally when developing the CNCC role were identified by participants as negative influences. Nursing knowledge and competence were considered undervalued and sub-optimally used.

#### Cluster 6: motivation and support for competence development

This cluster consists of participants' ideas structured around how competence and competence development have played a crucial role in moulding the CNCC role. Participants underscored how this cluster is particularly dependent on other clusters – not least cluster two – determining if CNCCs are able to access competence development activities, such as courses, conferences and network meetings. One idea concerned how advancements in cancer care have necessitated the development of the CNCC role, as care trajectories are increasingly complex and individualised. Furthermore, the drive and grit demonstrated by individual CNCCs in improving care were described as an important force.

#### Cluster 7: patients’ needs and position

Stakeholders emphasised that cancer patients’ needs and demand for improved quality of care, not least in terms of availability and continuity, have had a vast impact in shaping the CNCC role. The motivation to strengthen patients’ rights and position in healthcare can be described as an overarching trend, seen through legislation, the increased democratisation of healthcare and the rise of person-centred care, all of which have impacted why and how the CNCC role was established and its subsequent development.

### Synthesis of clusters

Actors at micro, meso and macro levels seem to have been able to function as gatekeepers or enablers in the implementation and development of the CNCC role, as the governance style and strategies that have been employed have emphasised non-mandatory and pragmatic approaches, rather than strict benchmarking or requirements. This route seems to have been a viable option to get the role implemented in the Swedish cancer care system, but findings indicate challenges in terms of equity, role clarity and resource allocation. Overarching issues concerning the view of nursing and the patients’ position in healthcare also seem to have permeated the process.

## Discussion

CM is an integrative method where insights are co-produced with stakeholders, but also interpreted by the researchers.^27^ In the following section, we will place the results in a scholarly and theoretical context and reflect on their implications. The result of the current study harmonises with previous research on advanced cancer nursing roles, while offering a novel perspective through the inclusion of diverse stakeholders and an emphasis on the structural realm of cancer care. Structural aspects of the organisation of healthcare have an undeniable influence on service quality. For instance, Cantril *et al*^18^ identified that unstandardised processes, unclear role delineation and insufficient resources hindered the implementation of oncology nurse navigator services in the US. Similarly, Kerr *et al*^22^ noted that the flexibility of the clinical nurse specialist role in cancer care may lead to misinterpretation and inappropriate use. In the Swedish context, studies^14–16^ have identified challenges related to limited support, unclear role definitions and inadequate working conditions.

The importance of the structural aspects of healthcare is, for example, reflected in the writings of Lachman *et al*^41^ and McCormac and McCance.^6^ The latter describe what they refer to as the *care environment* as significant for the enactment of person-centred care. Similarly, Lachman *et al*^41^ note that all aspects of quality of care must be reviewed from three angles: the receivers, the providers and the organisation. What this study adds is an illustration of the multidimensionality of the care environment or organisation, where influences at the micro (local) level interact bidirectionally with the meso- (regional) and macro-level factors (national or international governance structures). For example, Lachman *et al*^41^ argue that to achieve effectiveness (a core dimension of quality), evidence-based practices at the provider end must be matched with benchmarking of process and outcome indicators on the organisational level to truly attain high-quality care. As healthcare settings differ substantially, the study findings are not generalisable in the traditional sense, but serve as an illustration of the dependencies between structural domains. The results emphasise the responsibility and influence of actors across the entire organisation of healthcare services when implementing and developing advanced nursing services in cancer care.

Healthcare systems are complex and adaptive, and implementation and development processes in this context do not adhere to linear logics. Instead, they are defined by path dependency, contextual factors and social ties and interactions between system actors.^42^ The findings from the current study case can be reviewed through this lens: a national initiative in a decentralised healthcare system to be implemented across a variety of operations (oncology, urology, haematology etc.) without an outspoken strategy for implementation and follow-up that considered the complex adaptive nature of the system. The process seems to have been driven by a strong wish to improve patients’ processes and outcomes, but insufficient attention may have been paid to the structural realities of the context. The results of this study also indicate a lack of the ambidextrous use of both professional and improvement knowledge required to achieve improved outcomes.^25^

Additionally, we suggest that the notion of magic concepts can elucidate the findings further. The term emanates from public management research and refers to broad ideas and concepts that gain traction by promising to resolve persistent dilemmas through their apparent universality. They attract policymakers and leaders because they are difficult to oppose and seem to minimise conflict. However, they often remain overly abstract and risk becoming empty signifiers.^43 44^ Examples of magic concepts that have previously been discussed in the literature include ‘accountability’, ‘co-creation’ and ‘trust’. We propose that this can apply to the implementation and development of the CNCC role, representing a novel application of the term magic concepts to a professional role. The CNCC role is associated with high expectations of managing and neutralising complex and overarching challenges in cancer care, such as inequity, continuity and patient involvement. While these expectations have been realised in some contexts, in others, the role’s value remains constrained.^8^ A key challenge is that the CNCC role is not clearly distinct from other nursing roles regarding orientation or purpose. Rather, it reflects an intensification—more continuity, assessments and individualised support. This vertical differentiation is challenging to define and measure. Limited consensus and governance have rendered the role a somewhat abstract solution to complex problems, leaving CNCCs navigating tensions between patient needs, organisational conditions and high yet vague expectations. We thereby suggest that the CNCC role would benefit from becoming more formalised, in likeness with other advanced nursing roles in cancer care.

### Strengths and limitations

The strengths of the study include the novel methodological approach to the area of investigation. The research group have striven to ensure study validity and reliability throughout the process by thoroughly adhering to the CM methodology, carefully considering biases and including a broad range of stakeholders. Furthermore, the stress value of .27 indicates that the two-dimensional concept map represents the idea sortings well.

The study also has limitations. Purposeful criterion sampling was used to obtain insights from diverse and information-rich participants, intended to capture a broad range of perspectives.^37^ However, this means that the researchers determine which voices are included, and the breadth of perspectives in the wider population remains unknown, precluding claims of generalisability. Although the research reflected carefully on these issues, potential selection bias cannot be ignored.

The aim includes investigation of both the development and implementation of the CNCC role, while the prompt only includes the term ‘development’, as the research group considered this would be sufficient given how we perceive that word to be understood in everyday Swedish language. This claim was not supported through pilot testing or cognitive interviews, which constitutes a limitation of the study. However, the ideas generated by the stakeholders can be said to mirror both these concepts, indicating that the prompt was functional in this regard.

The total response rate (33%) in the idea generation phase was calculated based on the 248 people initially invited. This figure is shrouded in some degree of uncertainty, as a small number of respondents forwarded the invitation to colleagues. However, it is deemed an informative representation of the response rate. It was lower than anticipated, limiting representativeness. While CM prioritises recruiting appropriate participants over achieving large samples, variation in response rates across stakeholder groups (26–46%) constitutes a limitation. The underrepresentation of the local management and governance and the organised advocacy groups (both 26%) may reflect shortcomings in how the relevance of participation was communicated. It is possible that the method of recruitment through email is insufficiently captivating to engage participants in this type of study. This is also reflected in the uneven distribution of sorters in phase 3, where a majority represented the stakeholder group ‘Regional and national leadership and governance’. Nonetheless, the sample consisted of a wide variety of actors, representing different perspectives and experiences.

The AI-tool IdeaDistiller^38^ was used to support the structuring of ideas. Although IdeaDistiller has demonstrated validity for supporting statement synthesis, AI-assisted clustering may have influenced the initial grouping of semantically similar statements. To mitigate this limitation, all outputs were reviewed by the research team and all final decisions regarding statement reduction were made by the researchers. 58 ideas were excluded for falling outside the study scope—a relatively large proportion—which raises questions about the clarity of the prompt. Most of the ideas concerned perceived benefits of the CNCC role, particularly from participants in the ‘Organised advocacy groups*,’* with whom the prompt was not pilot tested. This perspective was captured elsewhere in the data, most notably in cluster seven, ensuring that the overarching sentiment remained represented.

The duration of the dialogue meeting was 2 hours. The tentative concept map was sent out via e-mail to participants beforehand. Given the amount of information and data to be discussed, the time allocation might be criticised for being too short. Yet a longer meeting might have made participation difficult for some participants. Furthermore, the study did not include a conventional individual rating procedure. Instead, a modified dialogue-based rating task was used during the interpretation phase. While this approach facilitated collective reflection and exploration of different perspectives, it does not provide the same level of quantifiable prioritisation as individual ratings and may have allowed certain views to receive greater attention during group discussions.

Finally, the study deviates from the ConMapT protocol by not including the sixth stage, ‘Utilisation,’ as the study aim was limited to the generation, structuring and interpretation of stakeholder perspectives. Consideration of how the findings may be applied in practice will therefore take place outside the scope of this study.

### Ethics

The study does not involve collection or processing of sensitive personal data, thus falling outside the scope of the Swedish Ethical Review Act (SFS 2003:460), not necessitating approval by the Swedish Ethical Review Board. However, the project plan and the participant information letter were reviewed by an ethics counsellor at Karlstad University before the data collection (Reg. no. HNT 2025/610).

Participants were provided with customary study information and gave their informed consent prior to participating. They were not compensated.

Data was only handled on platforms with adequate security, accessible solely to the research team. In accordance with Karlstad University’s preservation and culling plan for research material (Reg. no. 230/02), data will be preserved for at least 10 years, after which it can be selected for secure deletion.

## Conclusion

Efficient implementation and development of an advanced nursing role in cancer care require adequate prerequisites and consensus across several structural levels. Clarity and supportive leadership at the micro level need to be paired with access to resources, support and policies that align with the purpose and scope of the process. In the case of the CNCC role, an example of an emerging advanced nursing role in cancer care, the choices made regarding governance and management strategies—with a large element of flexibility and voluntariness—have opened many doors but might have had implications such as aggravating standardisation of care, evaluation and follow-up. Shedding light on how this process has shaped the CNCC role to date provides an important foundation for its continued development, offering guidance and insights for policymakers, managers and practitioners working to improve the quality of these services provided to individuals affected by cancer. Future research could beneficially investigate how different aspects of organisational prerequisites interact and influence each other to deepen the understanding of their impact on quality of care.

## Supplementary material

10.1136/bmjoq-2026-004573 online supplemental file 1

10.1136/bmjoq-2026-004573 online supplemental file 2

## Data Availability

Data are available upon reasonable request.
