# Supplementary material for: Understanding stakeholder perceptions of the implementation and development of an emerging advanced nursing role in cancer care: a Swedish concept mapping study
Source: BMJ Open Qual. 2026 Nov 6;15(4):e004573. doi: 10.1136/bmjoq-2026-004573 (PMC13756257; doi:10.1136/bmjoq-2026-004573)
Supplement: Supplementary file 1 [file bmjoq-15-4-s001.docx]

# **APPENDIX 1: CHECKLIST FOR REPORTING TROCHIMs CONCEPT MAPPING RESEARCH (ConMapT)**

| **Heading** | **Section and**  **topic** | **Item**  **#** | **Checklist item** | **Page** |
| --- | --- | --- | --- | --- |
| Title | Title | 1 | State that the manuscript is reporting a concept mapping study. | 1 |
| Abstract | Abstract | 2a | Describe the core problem (focus) of the research. | 2 |
|  |  | 2b | State concept mapping as the study methodology and list the phases of concept mapping^*^ undertaken. | 2 |
|  |  | 2c | Indicate where the fieldwork was conducted. | 2 |
|  |  | 2d | State the stakeholder groups involved in the research. Report the total number of participants involved in  the study. | 2 |
|  |  | 2e | Give a brief description of the final concept map that should include cluster labels. | 2 |
| Background | Rationale | 3 | Provide a sound scientific rationale for the study. | 4 |
|  | Aim | 4 | State the study aim(s). | 4-5 |
|  | Methodological justification | 5a | Provide a justification for using concept mapping to address study aim(s). | 5 |
|  |  | 5b | State which of the six phases^*^ of concept mapping were undertaken. Justify why phase(s) of concept  mapping were omitted. | 5 |
| Phase 1, Preparation | Determining the focus prompt | 6a | State the focus prompt for the study. | 6 |
|  |  | 6b | Describe the process for developing the focus prompt and, if applicable, how stakeholders were involved. | 6 |
|  | Participants | 7a | Justify why each stakeholder group was selected. | 6 |
|  |  | 7b | State the eligibility criteria for each stakeholder group. | 6 |
|  |  | 7c | Describe how study participants were recruited. | 6 |
|  |  | 7d | Provide a rationale for the number of participants at each phase of concept mapping. | 6 |
|  |  | 8a | Describe the flow of participants (ideally as a flow diagram) through each phase of concept mapping. | Fig 1 |
|  |  | 8b | Tabulate and describe the demographic and clinical (if relevant) characteristics of each of the stakeholdergroups. | 6 |
|  | Public  involvement | 9 | Explain how patients and/or the public were involved in each phases of the research (from conception  through dissemination). | 6-8 |
|  | Software  package | 10 | State which software package (e.g., Ariadne, Group wisdom [formally known as concept systems], Stata)  was used in each phase of concept mapping. | 7 |

| Phase 2, Generating the ideas | Brainstorming | 11a | State all methods used for idea generation (e.g., brainstorming, literature review). | 7 |
| --- | --- | --- | --- | --- |
|  |  | 11b | Describe in detail how brainstorming sessions were conducted (e.g., individual interviews, focus groups) and  recorded. | NA |
|  |  | 11c | Explain how data from the brainstorming sessions was turned into statements. | NA |
|  |  | 11d | Describe factors that may have influenced (e.g. group dynamics) idea generation. | NA |
|  |  | 11e | Report the number of statements generated from each approach to idea generation (literature review,  brainstorming). | 7 |
|  | Statement reduction | 12a | Describe and justify the procedure for statement reduction (e.g., eliminating overlapping statements, or  combining similar statements). | 7 |
|  |  | 12b | Describe to what extent participant wording was retained in the statements. | 7 |
|  |  | 12c | Describe how stakeholders were involved in statement reduction. | NA |
|  |  | 13 | Report the final number of statements following statement reduction. Ensure a complete list of the  statements is available (e.g., as a supplementary document or data file). | 7  Appendix 2. |
| Phase 3, Structuring the statements | Clustering and rating (or prioritisation**) | 14a | Describe the procedures followed for structuring statements (clustering and rating tasks). | 7 |
|  |  | 14b | Provide information about the instructions to participants as to how to complete the clustering and rating  tasks. | 7 |
|  |  | 15 | Report the average (mean, median) number of clusters generated by the participants. | 7 |
|  |  | 16 | Report the mean rating score for each statement (consider reporting as a supplementary document or data  file). | N/A |
| Phase 4, Concept mapping analysis | Data cleaning | 17a | Describe procedures for data checking and cleaning. | 7 |
|  |  | 17b | Describe procedures for handling missing data. | N/A |
|  | Statistical procedures | 18a | Specify what statistical procedures were undertaken to generate candidate concept maps. | 7-8 |
|  |  | 18b | Specify the statistical tests undertaken to determine the validity of the concept map (e.g., stress value, split-  half reliability test). | 7 |
|  |  | 18c | Describe any additional analyses not in the study protocol. | N/A |
| Phase 5, Interpreting the map | Data interpretation process | 19a | State how the final concept map was selected. | 7-8 |
|  |  | 19b | Provide a description of how cluster (on the final concept map) labels were determined. | 7-8 |
|  |  | 19c | Describe how stakeholders provided feedback on the final concept map. | 8 |
|  | Description of the final concept map | 20a | Describe the final concept map. | 8-10 |
|  |  | 20b | State and justify any post hoc adjustments to the concept map (e.g., moving statements between or combining clusters). | N/A |
|  |  | 20c | Describe each cluster (including illustrative statements) in the final concept map. | 8-10 Fig 2. |
|  | Rating data | 21 | If applicable, describe the go-zone (include examples of statements in each quadrant), or pattern match (ladder graph). | N/A |
| Phase 6, Utilization | Utilization | 22 | Provide a statement on how the concept map will be utilised. | 13 |
| Discussion | Discussion | 23 | Locate the concept map within the context of existing evidence. | 10-12 |
| Limitations | Limitations | 24 | State all study limitations (including those that may have arisen because of deviations from the study protocol). | 12-13 |
| Ethics | Ethics | 25a | State which ethics committee (or Institutional Review Board) reviewed the study (include the ethics committee reference number). | 13 |
|  |  | 25b | Describe the ethical issues in the study. | 13 |
|  |  | 25c | Describe the procedure for obtaining informed consent from the study participants. | 13 |
|  |  | 25d | State how participants were compensated for taking part in the study. | 13 |
| Conclusion | Conclusion | 26 | Summarise the key findings from the study. | 13 |
| Registration and protocol | Registration and protocol | 27a | Give details of the registration status of the study (including registry and registration number) | N/A |
|  |  | 27b | State how the study protocol can be accessed. | N/A |
|  |  | 27c | List any deviations from the study protocol. | N/A |
| ^*^ Six phases of concept mapping methodology, described by Trochim (Kane & Trochim, 2007) are 1. Preparation, 2. Ideas generation, 3. Structuring of statements, 4 Data analysis, 5. Interpretation, and 6. Utilization of maps;  ^**^ In some descriptions of concept mapping prioritising is described as rating.  **Please refer to the following citation for detailed information on the concept mapping process:**  Kane, M., & Trochim, W. (2007). *Concept mapping for planning and evaluation* (Vol. 50). Sage Publications. <https://doi.org/10.4135/9781412983730> | | | | |
