# Supplementary material for: Understanding stakeholder perceptions of the implementation and development of an emerging advanced nursing role in cancer care: a Swedish concept mapping study
Source: BMJ Open Qual. 2026 Nov 6;15(4):e004573. doi: 10.1136/bmjoq-2026-004573 (PMC13756257; doi:10.1136/bmjoq-2026-004573)
Supplement: Supplementary file 2 [file bmjoq-15-4-s002.docx]

# **APPENDIX 2: COMPLETE LIST OF UNIQUE IDEAS GENERATED IN THE STUDY^[1]^**

Cluster 1: Understanding and delineation of the role

1. that it is too focused on having ONE contact nurse, that is, based on a surgical investigation focus that does not fully match how we work in oncology where it is the team that is most important.

6. that the tasks correspond to the tasks of an oncology nurse and here their role has been overshadowed due to too much focus on the term ‘contact nurse’: you do not have to do the same thing for equal care.

9. that working as a contact nurse has not affected the salary, despite increased responsibility

14. that in many cases it has been coordinated with the role of standardised patient pathway coordinator

17. that there has been inflation in the role – when it exists in different operations, the possibility of having a coherent responsibility decrease

24. that the contact nurse role is a solution for larger operations/city-focused

26. that contact nurses are setto execute administrative tasks that should actually be performed by another profession

28. that regular outpatient nurses should be replaced

32. that the patient base is too large and contact nurses do not have time to support the patients

42. the increasing number of treatments

50. an unclear role description

61.the lack of structure for a career ladder

62. colleagues’ ignorance

63. the lack of competence, as nurses change workplaces more often today

68. uncertainty about whether the contact nurse replaces the responsible doctor

76. nurses who are resistant to change

79. that lack of time means that contact nurses are not allowed to attend training and do not have the opportunity to reflect on and develop the role

81. permissive or non-permissive colleagues

88. the lack of specialist nurses in oncology care

Cluster 2: Variance in resource allocation and prioritisation

2. that without organisational support and clear collaboration routines, the commitment risks becoming person-bound rather than systematic

19. that there was no unified picture: At the small hospital it was easy to say ‘we already work like this’, while university hospitals need this

27. that the time of the contact nurses for their work is prioritized in the clinics

39. the lack of organisational conditions for the assignment

40. the lack of, for example, oncologists and haematologists

47. inequality between units and hospitals when it comes to resourcing the contact nurse function

49. financial savings that reduce the conditions for performing their work fully

54. a hierarchy within the organisation

60. the introduction of new medical record systems that have taken time away from the assignment

65. that many testify that they do not get enough time for their assignment and are used for other tasks

80. that sufficient resources have not been allocated to carry out the assignment as a contact nurse according to the assignment description

Cluster 3: The importance of national governance

3. the new national mission statement that strengthens and elevates the role

10. the lack of national requirements for and follow-up on the implementation of the role

11. that the [national] cancer strategy is not sufficiently clear regarding the role of the contact nurse in cancer care

18. that it was included as an important assignment in the National cancer strategy in 2009

34. that the RCC^[2]^ has defined the role without anchoring it in healthcare, which means that the governance comes from the outside

35. that the RCC has participated in supporting and following up during the introduction of the role

45. the national working group for contact nurses within the RCC

46. the national role description that clarified the role

56. information, training and coordination through the RCCs

73. the creation and development of the RCCs

78. previous national guidelines from the National Board of Health and Welfare

87. the agreement between the state and the SKR^[3]^ with the mission and funding to design the contact nurse role

Cluster 4: The importance of local governance

4. that operational managers have seen the benefit of the contact nurse function

5. weak interest, non-existent listening and knowledge about the role in clinic management groups

7. that incentives need to be clarified for the regions to create the conditions for contact nurses.

12. that managers have little or no knowledge of the contact nurse role

22. that it has been up to the organisations to decide whether to introduce contact nurses

33. that money was invested in the project to introduce contact nurses, but no one checked that it was used for the intended purpose

44. the suspicion and negative view conveyed by managers in cancer care

51. commitment from both managers and employees

52. financing via an orderly introduction at an initial stage to establish the function

53. the freedom to describe the role based on a unit’s conditions

58. the lack of follow-up of the contact nurse role at the national, regional and local level

72. that the process of understanding the concept from [being] a strategy to [becoming] reality on the floor has taken a long time

77. the support or lack of support from managers and medically responsible people

83. the organisation’s ability to allocate time for nurses to establish and develop the role

Cluster 5: The legitimacy of nursing in the complex processes of cancer care

8. the use of existing evaluation systems to follow up and develop [care] via quality registers, PREM and the Classification of Health Care Measures.

16. that there is a national education plan for contact nurse education

20. that there is no formal competency requirement for specialist/contact nurse education, which has led to the role being interpreted in different ways despite the national mission statement.

21. that research has been published on the benefits of the role

23. that implementation should have been based to a greater extent on the evidence that existed from nurse-led clinics and advanced independent nursing roles

25. that the contact nurse is included as a central role in the National Clinical Cancer Care guidelines

29. that nursing is not always given the opportunity to develop

30. that nursing issues have received a clearer focus in the various national clinical care programmes

37. the need to have an overview and control in complex and sometimes fragmented cancer care

41. the role of the cancer coordinator

57. the introduction of ‘my care plan’ via 1177.se, which can facilitate the work as a contact nurse

71. the increase in the prevalence of cancer

74. regional nursing groups

75. the national association of nurses in cancer care

82. clearer work in diagnosis-specific care processes

85. the care programme for cancer rehabilitation that highlights rehabilitation needs

86. well-functioning team and process work

Cluster 6: Motivation and support for competence development

13. that they have received knowledge and training in development work

15. that the individualised care and treatments require a high level of competence from the contact nurse to coordinate, inform, etc.

43. the joint investment in training for contact nurses

59. the introduction of the health assessment instrument

64. the contact nurse’s versatile competence and ability to work in a person-centred way

66. opportunity to participate in network meetings and conferences and share/get inspiration from others

67. the opportunity to participate in/send contact nurses for further training

89. the contact nurses themselves through their drive and commitment

Cluster 7: Patients’ needs and position

31. that patient associations have pushed for each patient to have an assigned health care professional/contact nurse

36. that we have had to think more about nursing and work more individually according to patients’ wishes and needs

38. the need for continuity for cancer patients

48. the demand among cancer patients

55. how we view the patient and what needs the patient has

69. the patients right to safety, continuity and participation

70. patient representatives’ involvement in the issue for many years

84. the desire to improve [cancer care] for patients

1. Please note that the ideas have been numbered based on the order in which they were submitted.
2. Regional Cancer Centre
3. The Swedish Association of Local Authorities and Regions (SALAR)
